# Considerations for Embedding Inclusive Research Principles in the Design and Execution of Clinical Trials

**DOI:** 10.1007/s43441-022-00464-3

**Published:** 2022-10-14

**Authors:** Ubong Peters, Brenna Turner, Daniel Alvarez, Makaelah Murray, Aruna Sharma, Shalini Mohan, Shilpen Patel

**Affiliations:** 1 Product Development - Global Clinical Operations, South San Francisco, CA USA; 2 Genentech Inc., 1 DNA Way, South San Francisco, CA 94080 USA; 3 US Medical Affairs, South San Francisco, CA USA; 4 Global Program and Clinical Operations, Vaughan, ON Canada; 5 AstraZeneca, Cambridge, UK; 6 Global Medical Affairs, Washington, DC USA; 7 Gilead Sciences, Washington, DC USA

**Keywords:** Diversity, Health equity, Inclusive research, Clinical trials, Underserved minorities

## Abstract

There is a growing recognition that the clinical research enterprise has a diversity problem, given that many clinical trials recruit historically marginalized individuals or patients reflective of real-world data at a rate that is far below the incidence and prevalence of the disease for which the investigational therapy or device is targeting. This lack of diversity in clinical research participation can obscure the safety and efficacy of drug therapies and limits our collective ability to develop effective treatments for all patients, leading to even wider health disparities. This *review article* provides an in-depth analysis of the impact of this bias on public health, along with a description of some of the barriers that prevent historically marginalized populations from participating in clinical research. Some practical solutions that can be employed to increase diversity in clinical trial participation are also discussed, including the crucial role clinical trial sponsors, research organizations, patients, and caregivers need to play in supporting the industry to achieve this ambitious but necessary goal.

## Introduction

### Health Equity, Diversity and Inclusion in Clinical Trial Participation: What Does it Really Mean, and Why is it Such an Important Topic?

Health equity is a principle that seeks to reduce and ultimately eliminate unfair, unjust, unacceptable, and avoidable differences in healthcare that are linked to economic, social, or environmental disadvantage of various groups of people [1]. This term is often used to assess whether progress has been made toward achieving improved access and better health outcomes for previously underserved populations. Diversity embodies our individual differences which encompass the various ways we identify ourselves based on race and ethnicity, gender identity, age, socioeconomic status, national origin, ability status, and much more. Inclusion, on the other hand, describes a culture of acceptance and respect where all people and all groups feel a strong sense of belonging to the community, and are treated equitably in all facets of life.

A common misconception about the meaning of health equity, diversity, and inclusion is that it only pertains to Black and Brown individuals. Such thinking is misguided and works against the best interest of public health because there are many historically underrepresented communities around the world who would benefit from clinical trial participation. These communities include rural populations, people with low socioeconomic status, the elderly, children and adolescents, females, and differently abled people. Black and Brown individuals are also consistently underrepresented in clinical trials, as are American Indians, Native Hawaiians, Alaska Natives, and Asian and Pacific Islanders [2].

Clinical trials are an important part of the continuum of healthcare. These trials offer valid treatment options for many patients during the course of their care, essentially providing them with access to emerging therapies. Current estimates suggest that over 80% of clinical trial sites are concentrated in 25 high-income OECD (Organization for Economic Co-operation and Development) countries [3], even though developing countries represent the majority of the world’s population [4], and host nearly 90% of the worldwide burden of disease [5]. Surprisingly, disparities in access to clinical trials are also widely reported in high-income OECD countries, including the US [6, 7]. This calls for a more equitable distribution of access points to clinical trials, followed by affordable product access once approved by regulators, especially in communities with a high disease burden where that need is even more critical.

Clinical trials provide an opportunity to determine whether novel therapeutics and devices are safe and efficacious for everyone. However, due to a lack of diversity among clinical trial participants, it is sometimes not known whether these products will work for all people until it has been approved by regulators and widely used by the general public. It is well known that the mechanism of action of certain drugs does vary in different ethnicities, race, and gender groups [8–10]. A drug could fail to provide therapeutic benefit because it was tested in the wrong population, or it was tested and approved in a majority population that does not reflect the reality of the disease. For example, Black women have mortality rates for breast and uterine corpus cancers that are 41% and 98% higher, respectively, than non-Hispanic White women [11], despite the tremendous advancements in the development of novel therapeutics [12], and the steady improvements in overall cancer survival in the past three decades [13]. At first glance, this trend appears to be caused by socioeconomic factors such as lack of access to quality care, but an in-depth analysis revealed that the key studies of these cancer treatments were largely conducted on White women with little representation from Black women [14, 15]. Thus, it was not known at the time that the treatments being developed would have a lower efficacy profile in the Black patient population.

Many patients from underserved communities are inadvertently excluded from clinical trial participation because the criteria for exclusion refer to comorbidities, such as obesity, cardiovascular disease, and HIV, which are common in those communities [16, 17]. Yet, once the drug is approved, people with those comorbidities are often prescribed the treatment. How can we expect a therapy to work for all patients when those recruited to participate in the clinical trials are not fully representative of the patient population? The only way to predict whether novel therapeutics and devices will achieve the desired safety and efficacy profile in underserved minority groups is to have them participate in clinical trials.

**Figure 1:**
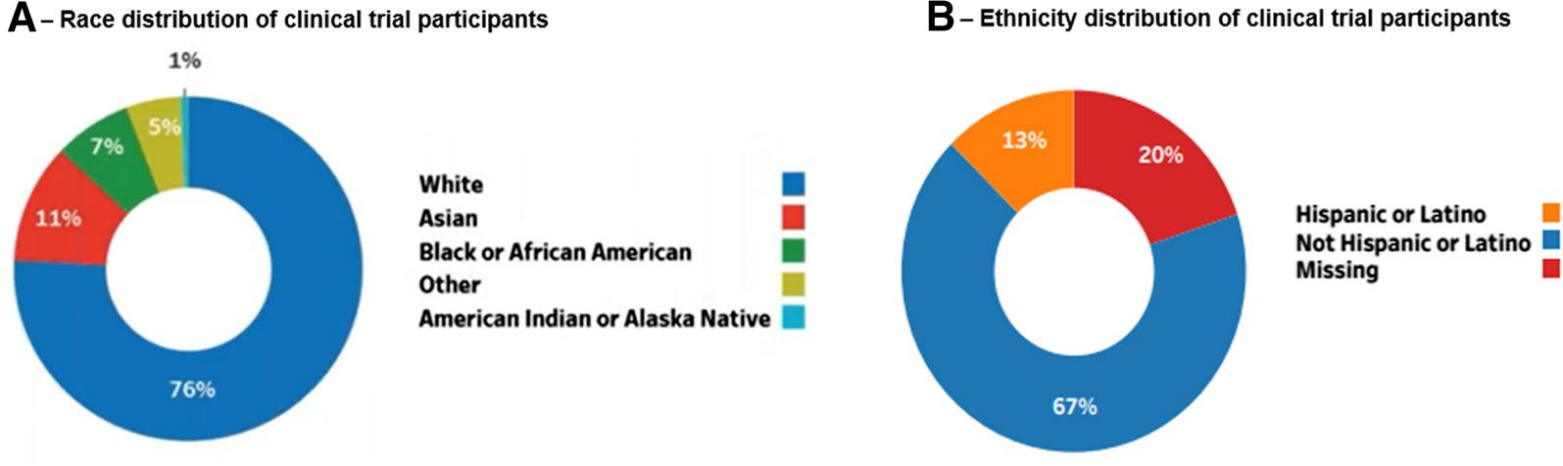
Demographics of Trial Participants in the 2015–2019 FDA Drug Trials Snapshot [18]. **A** Race distribution of clinical trial participants. **B** Ethnicity distribution of clinical trial participants.

**Figure 2:**
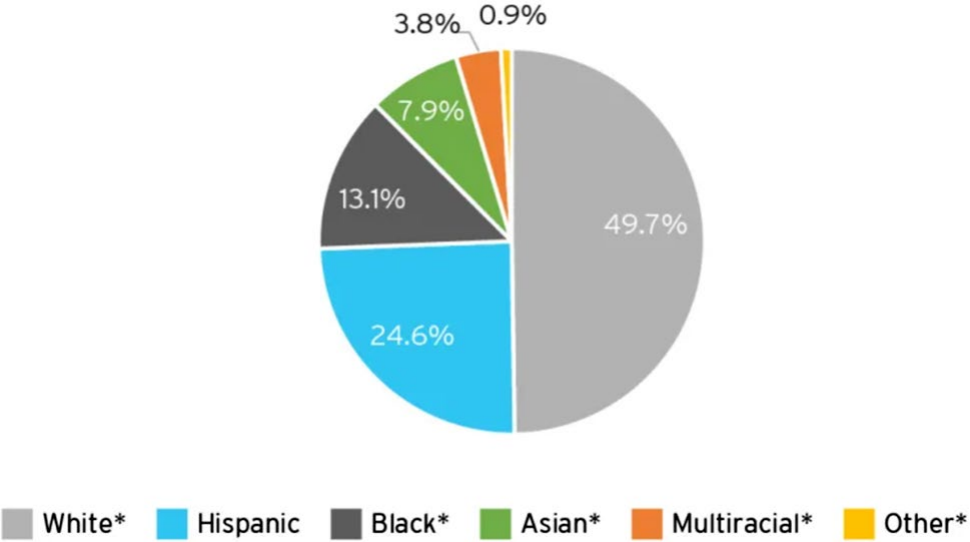
Projected racial profile of the US population by 2045 [20]. *Non-Hispanic members of race.

**Figure 3:**
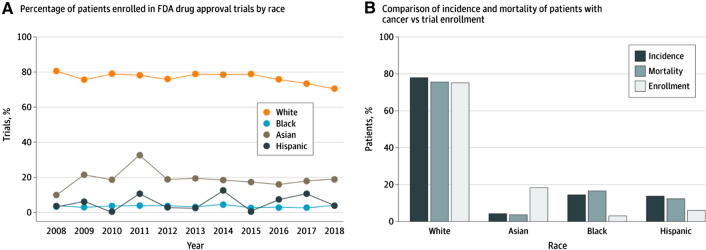
Disparity of race representation in clinical trials leading to cancer drug approvals from 2008 to 2018 [27]. **A** Proportion of different races in trials for FDA approval from 2008 to 2018. **B** Relative proportion of different races (pertaining to incidence and mortality) among patients with cancer in the US.

According to the 2015–2019 Food and Drug Administration (FDA) Drug Trials Snapshot (Fig. 1) [18], over 70% of clinical trial participants are White, whereas racial and ethnic minorities currently comprise almost 40% of the US population [19]. Interestingly, US population demographics are changing, and new census population projections suggest that by 2045, the US is likely to become “a majority-minority” nation (Fig. 2) [20]. Yet, more than 80% of genomic data that are driving the recent advances in personalized healthcare is derived from individuals of European ancestry [21–23], even though pharmacogenetic studies have demonstrated significant racial/ethnic differences in drug metabolism, safety, efficacy and other biomarkers [24–26]. The care standards developed with datasets that lack diverse representation stand the risk of not being generalizable, and this will lead to even wider disparities in healthcare. In oncology, there are already startling disparities in mortality and disease burden with all impact measures being worse in African Americans [11]. Yet, less than five percent of clinical trial participants in oncology are Black (Fig. 3) [27], even for trials focused on oncology indications, such as triple negative breast cancer (TNBC) that disproportionately affects Black women [28].

This bias limits our collective understanding of genetic and environmental causes of disease, and impedes population-wide efforts in disease prevention and treatment. More importantly, it prevents us from unlocking the potential of personalized healthcare, and optimizing treatment outcomes for all patients to achieve better diagnosis and better access to the right therapies. Thus, there is clearly a strong ethical, scientific and regulatory imperative to increase patient diversity in clinical research. From a scientific perspective, there is a paramount need to ensure that the populations treated in clinical trials match the populations that will eventually use the drug upon approval. This will promote a good understanding of any underlying differences in the drug’s mechanism of action that may be related to different racial or ethnic backgrounds and its attendant effect on the safety and efficacy profile of the drug [24–26].

There is almost unanimous agreement that health equity, diversity, and inclusion is a critical topic that must be considered and embedded in the design and execution of clinical trials. However, the prevailing perception is that it can be challenging to find the right balance between the desire to execute an inclusive clinical trial and the desire to move as quickly as possible to bring new treatments that improve the quality of life of patients suffering from a devastating disease. Given how cumbersome it can be to incorporate inclusive research principles in clinical trials, this topic is sometimes sidelined when we need to move quickly through the various stages of drug development. In such circumstances, we tend to rely on the traditional ways of doing things, using tried-and-true processes, and only engaging investigators and clinical sites we already know and trust. Although this is not an easy balancing act, we must reform our processes to target and recruit patients of diverse backgrounds into our clinical trials by carefully assessing and removing the barriers to participation, and intentionally incorporating some of the solutions described below.

## Barriers and Socioeconomic Factors That Cause Disparities in Trial Participation

In a 2019 study conducted by the Center for Information and Study on Clinical Research Participation (CISCRP) to elucidate the factors that affect clinical trial participation [29], 85% of the over 12,000 participants interviewed expressed a willingness to participate in clinical studies (Table 1). However, willingness does not always translate into participation, and the reasons can vary widely. While the purpose of the research, the risks, benefits, types of trial procedures, and compensation are all important factors that can influence the decision to participate in a clinical study, there are other key factors that could deter some people from participating. These include the potential costs of participation, mistrust of the healthcare system, location of the study site, study duration, and the number of study visits (Table 1). Here, we will describe some of the main barriers that lead to disparities in clinical trial participation.

**Table 1:** Factors that affect clinical trial participation. Adapted from the 2019 Perception and Insights Study conducted by the Center for Information and Study on Clinical Research Participation [29]

Key motivators*	Key barriers^†^
Advance science and treatments (62%)	Not wanting to risk health (49%)
Help others with my disease (57%)	Risks involved (46%)
Better treatment (51%)	Not knowing enough (25%)
Education about disease and treatment (47%)	Don’t want to be treated as a test subject (22%)
Compensation (42%)	Risk of receiving placebo (16%)
Access to healthcare providers (29%)	Too much time required (15%)
Free medication and treatment (28%)	Can’t afford time off (14%)
Information about the study (23%)	Too difficult to get to the research center (12%)
*Those who said they would be “Very willing” or “Somewhat willing” to participate (*n* = 10,479)	^†^Those who said they would be”Not at all” or “Not very willing” or “Unsure” about participating (*n* = 1974)

### Lack of Clinical Trial Sites in Underserved Communities

A lack of access to quality health facilities in underserved communities presents a barrier to participation in clinical research. The disproportionate burden of COVID-19 disease borne by racially/ethnically disadvantaged communities has revealed some undeniable truths about the inequities that exist in the US healthcare system today [30, 31]. The system has, by its very nature, failed to provide equal access to marginalized communities and patients of low socioeconomic status [32, 33]. This calls us to reimagine the entire paradigm that the drug development industry is built upon. Currently, clinical trial sponsors generally select the same type of clinical trial sites to run clinical trials. These sites tend to be large academic medical centers, or tertiary medical centers where the surrounding populations are rather homogenous and the catchment areas have low proportions of underrepresented patient groups. We have to take concrete steps to break that mold so we can take clinical trials to community healthcare centers where diverse and underrepresented patients live and get their healthcare delivered. This would provide more access points for patients from disadvantaged communities to participate in clinical trials.

### Mistrust of the Healthcare System

Even after patients from minority backgrounds who might benefit from clinical trial participation have been identified, they may not choose to participate in a trial. The patient must feel confident enough to volunteer to participate, and they must trust that the care they will receive will not put them in further harm. According to a 2017 study on the public and patient perception of clinical research [34], 28% of Black and 32% of White respondents report having little or no trust in pharmaceutical companies. While it is true that Black patients have a certain lack of confidence and trust in the healthcare system partly due to past injustices such as the Tuskegee experiment [35], it is also true that minorities are just as likely as Whites to participate in clinical trials [36]. This is consistent with the findings of a previous study of the enrollment decisions of over 70,000 individuals which reported that racial and ethnic minorities in the US are as willing as non-Hispanic Whites to participate in health research [37]. The hidden truth is that many clinical trial sponsors have not done a particularly great job at recruiting minority populations—they have not selected many researchers of color to participate as investigators in their clinical trials, even though these researchers may treat more underserved patients and may be more widely trusted in the community.

How can we fully address the issue of mistrust between patients and the healthcare system? One potential solution would be to train investigators to have a much more personalized interaction with the patient as opposed to a generic one. We should also broaden our investigator base to include clinicians and researchers who serve people of color because patients often feel more comfortable interacting with someone who shares and/or appreciates their cultural experience [38–40]. To properly address this particular barrier, we must also address the broader issue of lack of diversity in the clinical trial workforce. This will require a systemic solution that will take time to implement as it involves developing public–private partnerships, training the next generation of educators, medical practitioners, and the entire clinical research workforce while also diversifying the investigator base. We must work with private organizations in the communities where we identify disparities to educate local residents on clinical trials. We must also partner with academic institutions to launch mentorship programs to encourage and motivate the next generation of researchers, particularly those from underrepresented communities, to pursue careers in medicine and allied health professions, as well as STEM (science, technology, engineering, and mathematics).

### Inclusion/Exclusion Criteria and Other Study Design Factors

A study’s inclusion/exclusion criteria could summarily dismiss many patients from underserved backgrounds due to comorbid conditions such as high blood pressure, type 2 diabetes, obesity, and HIV+ status which may be more common in these communities [16, 17]. Additionally, age, weight, and health insurance type (or a lack of healthcare coverage) are oftentimes listed among the eligibility criteria even though they have nothing to do with safety, efficacy, and outcomes of the study. During clinical protocol development, study teams should use data analytics tools to carefully assess the impact each eligibility criterion will have on the inclusion and exclusion of underserved populations into the trial, and appropriate accommodations should be made as needed.

### Inadequate Reimbursement

As shown in Table 1, the out-of-pocket costs associated with clinical trial participation are one of the top barriers to enrollment, and reasonable compensation is a key driver for trial participation especially among participants from underserved communities [41]. While an offer of payment (reimbursement or compensation) is common practice in clinical research, some stakeholders and regulators are often concerned that such offers represent undue inducement (or coercion) [42], and may bias potential participants’ decision-making and compete with their self-interest and autonomy [43–45]. Interestingly, the FDA, the Office for Human Research Protections, the Council for International Organizations of Medical Sciences (CIOMS), and the Declaration of Helsinki do not prohibit payments to trial participants. In fact, these regulatory bodies, including the International Council for Harmonization of Technical Requirements for Pharmaceuticals for Human Use, explicitly support reasonable payment offers, so long as approval has been sought and obtained from the relevant Institutional Review Boards [46–48].

It is critical that we do not lose sight of the fact that many patients are unable to participate in a clinical trial because they cannot afford to take time off work, cannot afford childcare, or they live in a rural area that is far from the clinical site and cannot afford transportation. This is a broad socioeconomic issue that patients face which invariably affects their ability to participate in a clinical trial. We must, therefore, adopt a balanced approach toward safeguarding the participant’s autonomy, and protecting them from undue influence by the payment offer, while also paying thoughtful attention to the broader objectives of access to clinical trials, health equity, diversity and inclusion.

One systemic approach that should be considered is to ensure that patients who participate in clinical research do not have to bear any costs associated with their participation, because this would relieve the high burden placed on patients, particularly those that are economically disadvantaged. Payers, including Medicare and Medicaid, should be asked to cover out-of-pocket expenses of all patients participating in clinical trials, similar to the CLINICAL TREATMENT Act [49]. Clinical trial sponsors should also provide a mechanism for covering the healthcare expenses of clinical trial participants.

## Finding Solutions: What Should we do to Broaden Access and Achieve Diverse and Inclusive Representation in Trial Participation?

### Follow the Data

We have to be fully aware of the magnitude of this diversity problem in order to take appropriate action. Fortunately, there are many new analytical strategies and techniques that can help us understand all aspects of the disease, not just the top level information. First, we have to identify what the epidemiology of the particular disease is in a target population. And then, we have to find out what data are already available that suggests a disparate impact on the incidence, prevalence, severity, mortality, and risk factors of the disease, in order to identify conditions that we need to pay attention to. We have to maintain a catalog of target diseases for which there are disparate profiles relative to incidence, prevalence, severity, mortality, risk factors, concomitant treatments or the lack thereof in a particular population.

It is also important to characterize active and research-naive clinical sites located in regions of high disparity in order to elucidate the factors that are preventing research-naive sites from running clinical trials. The insights generated from such an exercise can be leveraged, along with other clinical operations tools, to support the initiation of trial activity at these sites. This would invariably provide many patients from underrepresented communities who could benefit from clinical trials an access point to participate.

Lastly, we must utilize business intelligence tools to generate overlays of disease burden and study sites, community practitioners, and hospitals where that disease burden exists, so we can be intentional in our effort to expand the clinical site footprint to recruit a more representative patient population. In addition, we must enrich our trials to match those at risk of getting the disease under investigation. It is important to be mindful that we don’t pursue diversity at the cost of inclusion. To avoid that problem, we should always develop a study diversity plan that matches with the epidemiology of that particular disease and its demographics, and periodically review enrollment data to determine whether those diversity targets are being met. As we look to the future, we must extend the leverage provided by real-world data and electronic health records to uncover new ways of utilizing the evidence that already exist in healthcare systems to generate deeper insights into healthcare disparities, with the ultimate goal of developing better analytical solutions.

### Expand Network of Clinical Sites

The recent advances in data analytics and technology have revolutionized clinical site selection practices, and the old-fashioned way of random site selection is now considered a relic of the past. Site selection and site development decisions should now be based on a strategic, data-driven framework that includes a rigorous assessment of epidemiological data, site startup timelines, historical enrollment data, and diversity data such as race/ethnicity, age, gender, and other social determinants of health measures.

Most patients reside in communities that are beyond the reach of traditional clinical trial sites, so we have to change our approach in order to reach these communities. We must look beyond traditional academic or tertiary medical centers as the primary anchor for research, and we must expand our network of clinical research sites into underserved communities by partnering and investing in community health centers and making them part of the research enterprise. This is really about repopulating the resources for those safety-net hospitals so they can be properly transformed into clinical research centers that have the necessary resources for clinical trial participation including research materials and equipment for conducting clinical trials, well-trained support staff, and IT infrastructure.

We should also expand our network of satellite sites in community hospitals and clinics to refer patients into clinical trials at the main clinical sites. Additionally, we must continue to evaluate whether the use of decentralized clinical trials, utilizing telemedicine, just-in-time site identification, mobile health and mobile health technologies can lower the barrier and increase access to larger numbers of patients for whom social determinants of health may limit their ability to participate in research. Finally, there is a broader role for the government to exert leadership here, and where that is absent, we should strongly advocate for the government to reform healthcare systems so everyone can have equal access to care.

### Broaden Study Eligibility Criteria

There is a misconception that the tighter we adjust the screws around the study protocol, the better the data quality will be. This is only true up to a certain point, beyond which it misrepresents the broader patient population affected by the disease. We have to rethink the research questions that we ask, and we have to redesign clinical trials to be more reflective of the standard of care that is provided in the real-world setting as opposed to relying on the standards of historical protocols. This requires a thoughtful examination of the inclusion and exclusion criteria and its impact on the ability to recruit patients from underserved communities, given the nature of the disease and the typical stage of diagnosis of the disease in minority communities. In that regard, we should utilize advanced analytics techniques to carefully examine the study protocol by comparing against the treatment guidelines and standard of care for each patient segment in order to identify parameters within the protocol that can be modified to broaden patient access while also ensuring patient safety.

### Integrate the Voices of the Patients and Caregivers into the Study Protocol

We should always reserve a seat at the table for patients, patient advocacy groups, and caregivers to be included as a central part of the research team. It is important to integrate the voices of the patients and their caregivers into the design of the study protocol. This integration can be achieved through patient steering committees, or by using the Patient Protocol Engagement Toolkit during protocol development and protocol review [50]. Such integration is critical in building trust with patient communities. Moreover, the patient’s perspective is invaluable in determining whether the planned study is portable, practical, and acceptable.

### Educate the Patient Care Communities on Health Equity, Diversity, and Inclusion

Even though there has been an increased dialog about health equity, diversity, and inclusion in the past few years, it is safe to assume that many important stakeholders have not yet joined the conversation. Part of that conversation involves educating the patient care communities on the topic [51], and also providing them with specific tools, resources, and agency to be able to effect change within their health systems. For example, the American Academy of Neurology (AAN) through its Diversity Working Group has created a cadre of Diversity Officers across Neurology departments throughout the US, and AAN supports them by providing tools to (i) educate the department on issues of health equity, diversity, and inclusion, (ii) evaluate some of the racist structures that may be present within their department, (iii) help clinical researchers in that department think through how they should be recruiting and retaining participants on clinical research trials. AAN is also developing a multi-faceted anti-racism core curriculum that is funded through the Health Equity Innovation Fund from Genentech [52]. This curriculum uses case studies to teach AAN members about the history of racism in the profession of Neurology. It also helps them to understand that the foundation of racism in the US is not just in healthcare, but also related to policing, housing policy, and other governmental policies, so all AAN members can appreciate how this potentially influences the patient. At the end of the curriculum, physicians learn to identify an area of racism within their workplace where they can work to change. These examples from AAN are worthy of emulation across other medical specialties.

### Conduct Regular Focused Grassroots Outreach Campaigns

Industry sponsors of clinical trials, academic institutions, and healthcare organizations should regularly host focused grassroots outreach campaigns to draw attention to the underrepresentation of racial and ethnic minorities in clinical trials. While these campaigns can be organized to broadly target the community in general, they should specifically be directed at patients, patient groups or patient advocacy groups, caregivers, and healthcare professionals, including physicians, medical students, physician assistants, nurse practitioners, clinical research coordinators, as well as regulatory agencies and local authorities.

These campaigns should engage the community through trusted voices, churches, and civil organizations. We should educate the groups on issues related to clinical trials, study design, and regulatory processes, so as to increase public confidence in the way clinical trials are conducted. We all have a role to play in helping the community to understand and appreciate the role clinical trials play in shaping and advancing the future of healthcare. There should be a sustained effort to partner and engage with the community on a regular and ongoing basis.

### Empower Sites to Assess Their Performance on Diversity and Effect Change

In recent years, the clinical research industry has developed highly sophisticated tools for extracting valuable insights from clinical sites—from the specialty of the investigators at each site, to the historic enrollment performance of sites. However, the performance of sites on diversity and other cultural competency indices remains an important blind spot in our analyses. Recently, a new survey tool which aims to shine a light on this blind spot has been launched by the Society for Clinical Research Sites (SCRS). The survey tool—known as the Diversity Site Assessment Tool—is designed to help sites assess their performance on diversity [53], so they can determine what resources, training and additional support are needed to improve the site’s performance on diversity and cultural competency. For example, some investigators or study coordinators may be unable to spend the time required with patients to explain the clinical trial and answer any questions patients may have. There may also be cultural myths or just lack of understanding or language barriers. Clinical trial sites should employ dedicated staff members that speak the patient’s language and understand the patient’s cultural background to support patients in navigating trials conducted at the site.

### Diversify the Clinical Trial Workforce

Clinical trial sites should commit to recruiting investigators, nurses, and study coordinators from diverse backgrounds so patients can see themselves in the healthcare workforce. Clinical trial sponsors should amplify support for the development of clinical naïve-investigator programs where prospective investigators, nurses, study coordinators, and site monitors of diverse backgrounds can be trained on clinical research to pave a pathway for diversifying the clinical trial workforce. One such effort is underway by the Association for Clinical Research Professionals (ACRP) [54]. ACRP recognizes that a more diverse clinical trials workforce will have a direct positive correlation with more diverse clinical trials.

### Collaborate with Other Organizations to Systematically Tackle the Diversity Problem

There is a critical need for all relevant stakeholders across various organizations to come together as one to create a unified sustainable model that allows all parties to collaborate with a unified purpose. Previously, various organizations including the Multi-Regional Clinical Trials Center, Collaborative Institutional Training Initiative Program, Clinical Trials Transformation Initiative, Association of Clinical Research Organizations, CISCRP, FDA, ACRP, and TransCelerate BioPharma Inc have tackled the diversity problem in their respective siloes. However, the healthcare industry needs to develop a comprehensive strategy—a beacon that holds and unifies the efforts of all interested parties. We have to pull everybody who needs to be at the table together to come up with a holistic plan, connect the dots, and think outside of the box, so we can solve this diversity problem once and for all, and also deal with the inequities that have been inherent in our healthcare system for way too long. This would herald a new era of cooperation where there would be no siloes in our investments to address this issue.

### Implement Decentralized and Hybrid Clinical Trial Approaches

Decentralized or hybrid models of clinical trials have the potential to remove a major logistical barrier to clinical trial participation by creating a flexible model that is focused on a unique patient’s needs. Decentralized or virtual clinical trials integrate digital data collection, digital monitoring, telemedicine, and mobile (home) nursing to minimize the number of on-site visits required during the conduct of clinical trials. Hybrid trials integrate a combination of decentralized approaches with traditional clinical trials.

The value of these decentralized models became more apparent during the height of the COVID-19 pandemic where they supported remote patient management and optimized patient experience and engagement. These decentralized approaches support both remote and on-site trials in order to increase access to clinical trials, support patient recruitment, and increase enrollment and retention.

Technology solutions that allow sponsors and CROs to conduct decentralized and/or hybrid clinical trials must ideally incorporate elements of telemedicine, eConsent, electronic clinical outcome assessment (eCOA), electronic patient-reported outcome (ePRO), and eSource or electronic source data which is defined by the FDA as data initially recorded in electronic format.

eCOA is an electronic clinical outcome assessment that measures and records how patients feel and function during clinical trials to measure the efficacy of a health intervention. It is often used in conjunction with other quantitative clinical data collection methods to assess the patient’s experience. eCOAs are deployed through various data collection technologies that allow patients to remotely report information related to healthcare outcomes through handheld devices, tablets, computers, and interactive voice response systems.

eCOA is an umbrella term that covers a wide range of outcomes captured from clinical trials, including clinical outcomes reported by a healthcare professional, observer-reported outcomes reported by a caregiver, patient-reported outcomes (PRO), and ePRO. eCOAs enhance the operational efficiencies of clinical trials, providing trial participants with the flexibility of completing trial questionnaires and assessments from a convenient location such as at home, office, or clinic. A reduction in the number of required site visits could invariably increase the accessibility of clinical trials and improve patient retention.

Likewise, digital transformation is also revolutionizing the informed consent process. Consent forms are often too lengthy and difficult to comprehend, thus making clinical trials inaccessible to the lay public, and reducing patient retention. Furthermore, the informed consent process typically takes place during an in-person visit for patients who are within travel distance to the site. eConsent removes these barriers by incorporating interactive elements within the consent forms that can be tailored to suit audiences of various backgrounds. These include hyperlinked terms or glossaries for additional contexts, infographics and pictures, as well as audio and video elements that can be translated into a patient’s or caregiver’s native language. Since the eConsent form is tailored to the patient’s learning style, the result is a simplified and consistent electronic document that is engaging, interactive, and easy to understand. In addition, eConsent facilitates the remote conduct of the consent discussion, thereby extending trial access to a geographically diverse population of patients, while simultaneously reducing the screening period. Clinical trial sponsors should ensure they allocate sufficient funds in their trial budgets to adopt these innovative solutions and drive the change in mindset needed to embed these tools in future clinical trials.

## Conclusion

Achieving diversity in clinical trial participation is a difficult, time-consuming, and expensive exercise. While the data presented here focused mainly on the US, this discussion is not exclusive to the US—it is a global issue, and there are many underrepresented communities around the world that rely on us to do better. This is a systemic problem, and systemic problems require systemic solutions. As such, we have to rethink and redesign the systemic framework for clinical trial execution to incorporate various multi-faceted approaches that will help lower the barriers and increase access for underserved communities to participate in clinical trials. When we consistently enroll representative populations in all our clinical trials, we will collectively gain a much better understanding of the safety and efficacy profiles for the drugs we develop which will benefit all patients.

## References

[1] Braveman P (2014). What are health disparities and health equity? We need to be clear. Public Health Rep.

[2] Rubin E. Striving for diversity in research studies. *N Engl J Med*. 2021; 1429–1430. 10.1056/NEJMe2114651 34516052

[3] Drain PK, Parker RA, Robine M (2018). Global migration of clinical research during the era of trial registration. PLoS ONE.

[4] United Nations, Department of Economic and Social Affairs, Population Division. *World Population Prospects: The 2015 Revision, Key Findings and Advance Tables*. https://population.un.org/wpp/Publications/Files/Key_Findings_WPP_2015.pdf.

[5] Murray CJL, Lopez AD, Bryant JH, Harrison PF (1996). Global comparative assessments in the health sector: disease burden, health expenditures, and intervention packages. Global health in transition: a synthesis: perspectives from international organizations.

[6] Seidler EM, Keshaviah A, Brown C (2014). Geographic distribution of clinical trials may lead to inequities in access. Clin Investig.

[7] Feyman Y, Provenzano F, David FS (2020). Disparities in clinical trial access across US urban areas. JAMA Netw Open.

[8] Roden DM, Wilke RA, Kroemer HK (2011). Pharmacogenomics: the genetics of variable drug responses. Circulation.

[9] Yasuda SU, Zhang L, Huang S-M (2008). The role of ethnicity in variability in response to drugs: focus on clinical pharmacology studies. Clin Pharmacol Ther.

[10] Ahmed S, Zhou Z, Zhou J (2016). Pharmacogenomics of drug metabolizing enzymes and transporters: relevance to precision medicine. Genomics Proteomics Bioinform.

[11] American Cancer Society. *Cancer Facts & Figures for African Americans 2019–2021*. https://www.cancer.org/content/dam/cancer-org/research/cancer-facts-and-statistics/cancer-facts-and-figures-for-african-americans/cancer-facts-and-figures-for-african-americans-2019-2021.pdf.

[12] Liu J, Pandya P, Afshar S (2021). Therapeutic advances in oncology. Int J Mol Sci.

[13] Siegel RL, Miller KD, Jemal A (2020). Cancer statistics, 2020. CA Cancer J Clin.

[14] Food and Drug Administration. *Drug Trial Snapshot: TRODELVY*. https://www.fda.gov/drugs/drug-approvals-and-databases/drug-trial-snapshot-trodelvy.

[15] Food and Drug Administration. *Drug Trials Snapshot: ENHERTU*. https://www.fda.gov/drugs/drug-approvals-and-databases/drug-trials-snapshot-enhertu.

[16] Jadad AR, To MJ, Emara M (2011). Consideration of multiple chronic diseases in randomized controlled trials. JAMA.

[17] Boyd CM, Vollenweider D, Puhan MA (2012). Informing evidence-based decision-making for patients with comorbidity: availability of necessary information in clinical trials for chronic diseases. PLoS ONE.

[18] Food and Drug Administration. *2015–2019 Drug Trials Snapshots: Summary Report*.; 2020. https://www.fda.gov/media/143592/download.

[19] Frey WH. The nation is diversifying even faster than predicted, according to new census data. *Brookings*. 2020. https://www.brookings.edu/research/new-census-data-shows-the-nation-is-diversifying-even-faster-than-predicted/.

[20] Frey WH. The US will become “minority white” in 2045, Census projects. *Brookings*. 2018. https://www.brookings.edu/blog/the-avenue/2018/03/14/the-us-will-become-minority-white-in-2045-census-projects/

[21] Devaney S (2019). All of us. Nature.

[22] McGuire AL, Gabriel S, Tishkoff SA (2020). The road ahead in genetics and genomics. Nat Rev Genet.

[23] Mills MC, Rahal C (2019). A scientometric review of genome-wide association studies. Commun Biol.

[24] Ortega VE, Meyers DA (2014). Pharmacogenetics: implications of race and ethnicity on defining genetic profiles for personalized medicine. J Allergy Clin Immunol.

[25] Burroughs VJ, Maxey RW, Levy RA (2002). Racial and ethnic differences in response to medicines: towards individualized pharmaceutical treatment. J Natl Med Assoc.

[26] Shastry BS (2005). Pharmacogenetics and the concept of individualized medicine. Pharmacogenomics J.

[27] Loree JM, Anand S, Dasari A (2019). Disparity of race reporting and representation in clinical trials leading to cancer drug approvals from 2008 to 2018. JAMA Oncol.

[28] Schmid P, Adams S, Rugo HS (2018). Atezolizumab and Nab-Paclitaxel in advanced triple-negative breast cancer. N Engl J Med.

[29] Center for Information and Study on Clinical Research Participation. *2019 Perceptions and Insights Study*. https://www.ciscrp.org/wp-content/uploads/2019/12/Deciding-to-Participate-04DEC-1.pdf.

[30] Sze S, Pan D, Nevill CR, et al. Ethnicity and clinical outcomes in COVID-19: a systematic review and meta-analysis. *eClinicalMedicine*. 2020;29:100630. 10.1016/j.eclinm.2020.100630 PMC7658622 33200120

[31] Vasquez RM (2020). The disproportional impact of COVID-19 on African Americans. Health Hum Rights.

[32] Becker G, Newsom E (2003). Socioeconomic status and dissatisfaction with health care among chronically ill African Americans. Am J Public Health.

[33] Anderson NB, Bulatao RA, Cohen B, National Research Council (2004). Critical perspectives on racial and ethnic differences in health in late life. Panel on race, ethnicity, and health in late life.

[34] Center for Information and Study on Clinical Research Participation. *General Perceptions and Knowledge on Clinical Research*. https://www.ciscrp.org/wp-content/uploads/2019/06/2017-CISCRP-Perceptions-and-Insights-Study-Perceptions-and-Knowledge.pdf.

[35] Jones JH, Tuskegee Institute. *Bad blood: the Tuskegee syphilis experiment*. New York; London: Free Press; Collier Macmillan Publishers; 1981.

[36] Katz RV, Lee Green B, Kressin NR (2007). Willingness of minorities to participate in biomedical studies: confirmatory findings from a follow-up study using the Tuskegee Legacy Project Questionnaire. J Natl Med Assoc.

[37] Wendler D, Kington R, Madans J (2006). Are racial and ethnic minorities less willing to participate in health research?. PLoS Med.

[38] Campbell A, Sullivan M, Sherman R (2011). The medical mission and modern cultural competency training. J Am Coll Surg.

[39] Yao CA, Swanson J, McCullough M (2016). The medical mission and modern core competency training: a 10-year follow-up of resident experiences in global plastic surgery. Plast Reconstr Surg.

[40] Chipidza FE, Wallwork RS, Stern TA (2015). Impact of the doctor-patient relationship. Prim Care Companion CNS Disord..

[41] Fisher JA, McManus L, Kalbaugh JM (2021). Phase I trial compensation: how much do healthy volunteers actually earn from clinical trial enrollment?. Clin Trials.

[42] Williams EP, Walter JK (2015). When does the amount we pay research participants become “undue influence”?. AMA J Ethics.

[43] Largent EA, Emanuel EJ, Lynch HF. Filthy lucre or fitting offer? Understanding worries about payments to research participants. *Am J Bioethics*. 2019; 1–4. 10.1080/15265161.2019.1631076 31543034

[44] Grady C (2001). Money for research participation: does it jeopardize informed consent?. Am J Bioethics.

[45] Resnik DB (2001). Research participation and financial inducements. Am J Bioethics.

[46] Food and Drug Administration. *Payment and Reimbursement to Research Subjects*. https://www.fda.gov/regulatory-information/search-fda-guidance-documents/payment-and-reimbursement-research-subjects.

[47] International Council for Harmonization of Technical Requirements for Pharmaceuticals for Human Use (ICH). *Integrated Addendum to ICH E6(R1): Guideline for Good Clinical Practice E6(R2)*. https://database.ich.org/sites/default/files/E6_R2_Addendum.pdf.

[48] Council for International Organizations of Medical Sciences (CIOMS). *International ethical guidelines for health-related research involving humans*. https://cioms.ch/wp-content/uploads/2017/01/WEB-CIOMS-EthicalGuidelines.pdf. 40523065

[49] Lujan BR [d-N-3]. CLINICAL TREATMENT Act. H.R.913. https://www.congress.gov/bill/116th-congress/house-bill/913.

[50] Elmer M, Florek C, Gabryelski L (2020). Amplifying the voice of the patient in clinical research: development of toolkits for use in designing and conducting patient-centered clinical studies. Ther Innov Regul Sci.

[51] Ioannidis JPA, Powe NR, Yancy C (2021). Recalibrating the use of race in medical research. JAMA.

[52] American Academy of Neurology (AAN). *AAN awarded grant for anti-racism training for neurologists*. https://www.aan.com/PressRoom/Home/PressRelease/4870.

[53] Foster D (2020). The diversity site assessment tool (DSAT), reliability and validity of the industry gold standard for establishing investigator site ranking. Integr J Med Sci..

[54] Association of Clinical Research Professionals (ACRP). *ACRP partners in workforce advancement: grow & diversify the workforce*. https://acrpnet.org/acrp-partners-in-workforce-advancement/acrp-partners-in-workforce-advancement-grow-the-workforce/.

